# Application of a computer-controlled adsorber/desorber system to monitor mercury in air or gas samples: Part 1. Calibration and system description

**DOI:** 10.1155/S1463924689000489

**Published:** 1989

**Authors:** Les Ebdon, Warren T. Corns, Peter B. Stockwell, Paul M. Stockwell

**Affiliations:** 1 Plymouth Analytical Chemistry Research Unit Department of Environmental Sciences Polytechnic South West Drake Circus, Devon Plymouth PL4 8AA UK; 2 P S Analytical Ltd Arthur House B4 Chaucer Business Park Watery Lane, Kemsing, Kent Sevenoaks TN15 6QY UK


Journal of Automatic Chemistry Vol. 11, No. 6 (November–December 1989), pp. 247–253

### Application of a computer-controlled adsorber/desorber system to monitor mercury in air or gas samples: Part 1. Calibration and system description

**Les Ebdon, Warren T. Corns**
*Plymouth Analytical Chemistry Research Unit, Department of Environmental Sciences, Polytechnic South West, Drake Circus, Plymouth, Devon PL4 8AA, UK*

**Peter B. Stockwell and Paul M. Stockwell**
*P S Analytical Ltd, Arthur House, B4 Chaucer Business Park, Watery Lane, Kemsing, Sevenoaks, Kent TN15 6QY, UK*

### Introduction

The analysis of mercury at low levels in the environment is of increasing significance. The application of the PSA Merlin Fluorescence Detector System has previously been described briefly for the analysis of low levels of mercury in water and air samples. The fluorescence approach for mercury analysis offers a wide linear dynamic range, simplicity of design concepts and the benefits of full automation [1]. The availability of inexpensive, powerful and versatile IBM-compatible computer systems and interfacing boards means that it is possible rapidly to set up fully automatic operating systems at the user's sites. For water analysis the addition of an autosampler and chemical reaction system to release mercury from the solution, coupled to the Merlin Fluorescence Detector using TouchStone software (manufactured by P S Analytical Ltd, Kemsing, Kent, UK), offer an automatic, routine measurement system which will measure mercury reliably down to a level below 0·020 ppb. The mercury produced by the reaction system, or vapour generator, is sparged for the liquid system in a gas/liquid separator and transferred to the Merlin Detector using a specific gas interface.

A simple flow of mercury entrained in argon passes the mercury source, and the fluorescence produced is measured on a conventional photomultiplier tube device. Argon provides the optimum sensitivity and, because the mercury line absorbs and fluoresces at the same wavelength, ensures maximum sensitivity. The performance of this simple system far exceeds that of conventional absorption systems, even when gold concentration systems are used.

The sensitivity of the Merlin Fluorescence Detector is such that it is possible to measure levels of mercury directly in air that is passed into the system. However, for many applications the level of mercury is quite low and some means of trapping the mercury prior to revaporizing into the detector must be used. Many gold trapping systems have been developed, based on gold shavings, platinum, gold mesh and, more recently, on the application of a gold-sand impregnated trap. The latter was developed by Temmerman and co-workers at the University of Ghent in Belgium [2]. Air is drawn over the trap at a set flow rate for a time and the impregnated sand traps the low levels of mercury in the air. On completion of the collection, the mercury is revaporized into a detector system. This provides a rapid means of quantifying mercury in air, but it can be further significantly improved by firstly using the Merlin Fluorescence Detector, and, secondly, by flushing out all the air with argon to obtain maximum overall sensitivity.

The device described here automates collection and revaporization, along with the replacement of air by argon. In addition, a fully automated collection and quantification system has been developed in association with the TouchStone IBM-compatible software. An absolute calibration procedure has also been developed by Dumarey *et al.* [3]; this is a simple, but effective, means to provide a primary standard, which is more effective than diffusion tube approaches and considerably more economical. This approach relies on the vapour pressure of mercury at a set temperature being accurately known. Calculation procedures are therefore integrated with the software so that on knowing the temperature and injected volume, a fixed definable quantity of mercury is adsorbed onto the gold-sand trap. This volume is subsequently revaporized into the detector and is measured both by peak height and peak area.

### Equipment used

A fully automated system to measure the mercury level in gas, to calibrate the system absolutely and to calculate the correct result requires the PSA Galahad Adsorber/Desorber linked to the Merlin Fluorescence Detection system. Each of these units is then connected to an IBM AT-compatible computer through a U-Micro DIO card, (U-Micro Computers, 12 Chetham Court, Calver Road, Winwick Quay, Warrington WA2 8RF, UK). The software developed by SpinOff Technical Systems (1 The Avenue, Hadleigh, Benfleet, Essex SS7 2DJ, UK), in association with P S Analytical, provides the control sequences and data collection to automate the procedures.

0142–0453/89 $3.00 © 1989 Taylor & Francis Ltd.

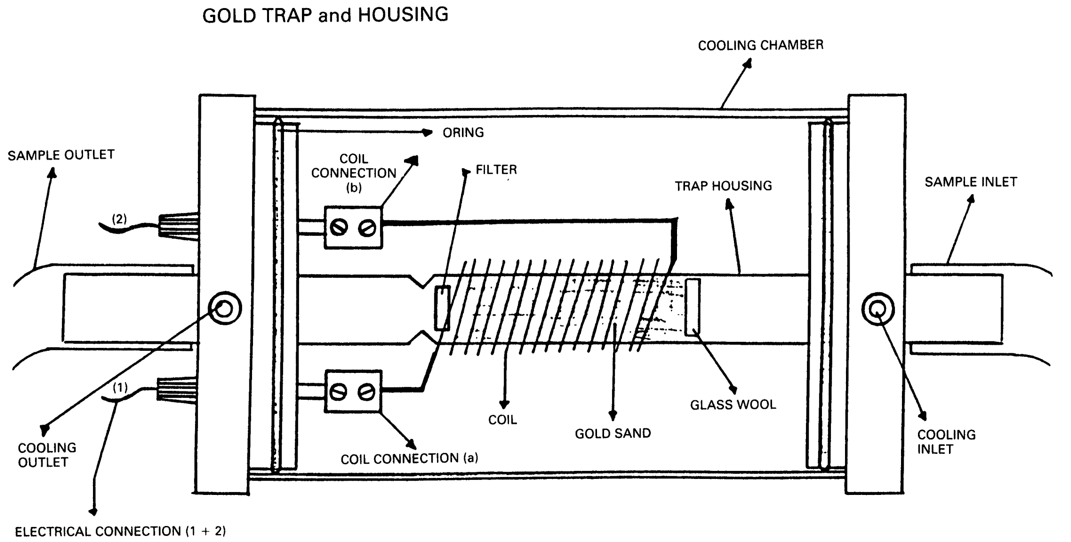

*Figure 1. Schematic arrangements of the gold-sand trap and heater assembly for Galahad; coil should run over the extent of the trap and filters etc. Mercury as a standard or sample is impregnated onto the sand. To revaporize the mercury the Galahad system is activated and its sequence commenced.*

### Adsorber/desorber system

The heart of the PSA Galahad is the gold-sand trap and heating system. A schematic diagram of this unit is shown in figure 1. The transport gas is swept through the trap, incorporating filters and retention glass wool at a cool temperature. Around the trap is a Nichrome coil, which is in turn connected to the power supply. Both the coil and trap are then retained in a specially designed cooling chamber. This allows visual inspection of the coil and trap and allows the cooling gas to pass over the coil to reduce temperature rapidly and allow the next sample to be collected by the Galahad. The cooling gas flow of either nitrogen or argon is simply vented to waste in the Galahad unit. With careful attention to cleanliness, the trap should have an extremely long life.

Three timed cycles are provided to operate once the mercury has been collected onto the gold sand. This collection can be either from a vapour generator, such as the PSA 10·003 Vapour Generator; a simple batch device, such as the Perkin Elmer MS20; or from samples of air or gas continuously fed over the trap. On completion of the collection, the Galahad is activated and the first timed delay is activated; this can vary from ½ to 2 min. This is used to stablize conditions and the argon flow is diverted through the trap to flush out residual air or gas. On completion, the heating cycle is commenced and the coil rapidly heated by an electric current to red heat. Three variable heating times are provided: 5, 10 and 20 s. This cycle rapidly vaporizes the mercury, which is then swept into the mercury device, for example into the PSA Merlin 10·023 Fluorescence Detector. Once the vaporizing stage has been completed, the heating coil and gold-sand trap should be cooled by the release of argon or nitrogen over the coil at a sensible flow rate. On completion of the third stage the cycle is complete and the trap is ready for re-use.

The Galahad can be activated either from the instrument panel, or, remotely, via a 15-Way D-Connector at the rear of the unit. With the TouchStone software, the Galahad Adsorber/Desorber for air or gas analysis occupies the interface used by the Vapour Generator or FIA valve. In this manner, a range of measurements can be made simply by substituting the appropriate modules.

The PSA 10·511 Galahad incorporates a valving manifold which diverts the gas and argon flow over the trap.

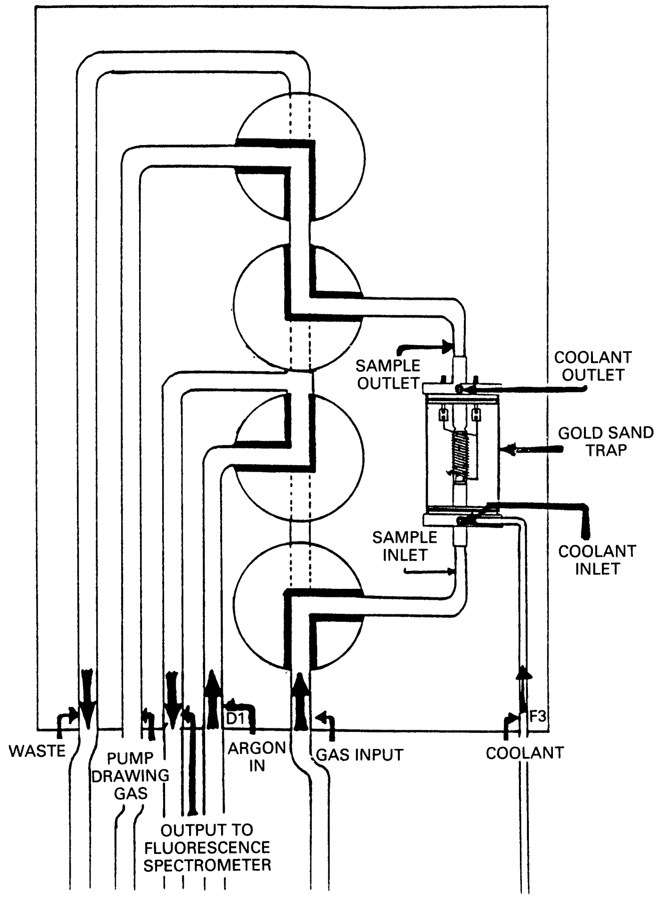

*Figure 2. Schematic layout of the valving assembly in the PSA 10·511 Galahad for adsorption/desorption.*

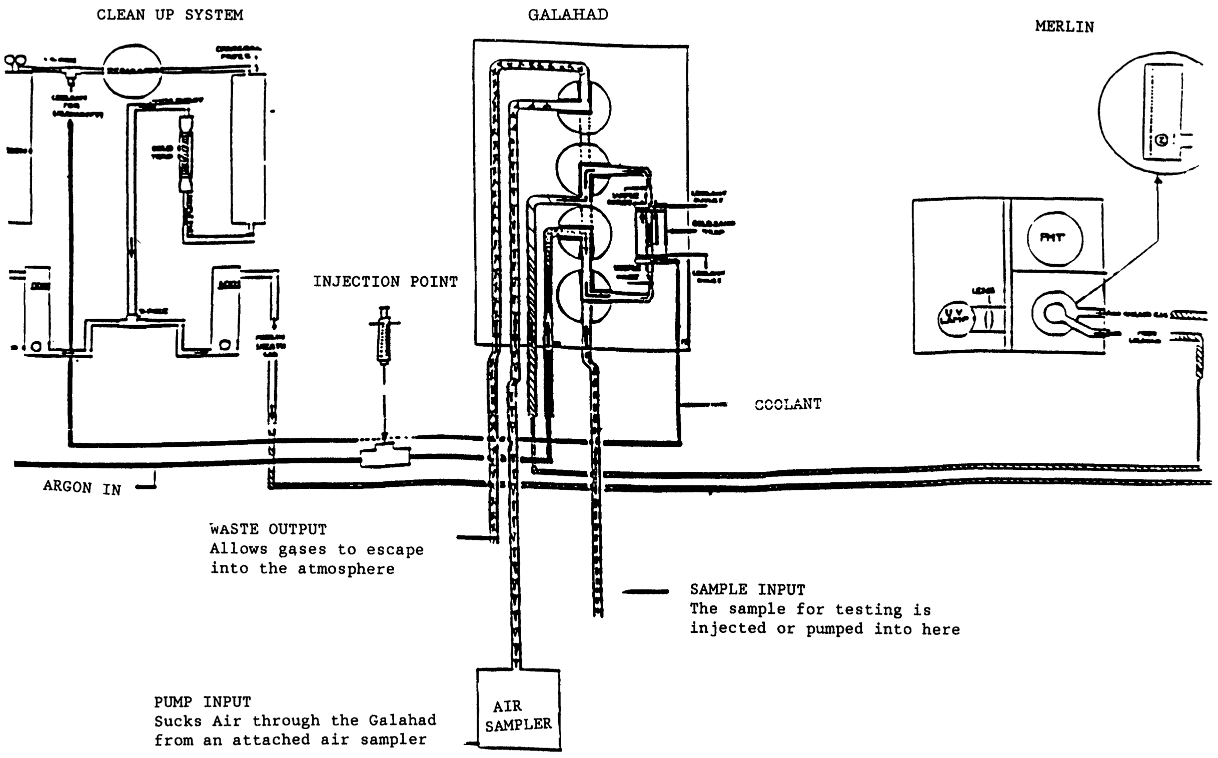

*Figure 3. Galahad configuration for calibration.*

Whilst the Galahad is not activated, an air or gas flow can be continuously passed over the gold-sand trap. In this way, if the flow rate and time for collection are known, it is possible to calibrate the concentration of the mercury in a unit volume of air or gas. During the collection stage an argon stream is fed directly to the detector, for example the PSA Merlin. The flow pattern is shown in figure 2, all the valves and flow paths are constructed from inert materials, i.e. PTFE valves and tubing.

Four Neptune Valves (Neptune Research Inc., 6 Lombardy Place, Maplewood, New Jersey 07040, USA) are incorporated into the manifold. All connections are of silicone tubing or PTFE couplings designed for the purpose.

Once the Galahad cycle is activated, the valve configuration changes so that the argon flow is diverted over the gold-sand trap. Air or gas is flushed out in the first stage of the Galahad cycle and conditions in the detector stabilized. As the mercury is revaporized, it is flushed into the detector and a response is produced.

### Fluorescence detector

The fluorescence detector measures mercury as cold vapour; mercury transported into the unit in a stream of argon gas is detected accurately over a wide linear dynamic range (0–2 ppm). The detector has been specifically designed to operate with detection levels better than 0·02 ppb. It operates on a quantized feedback ratiometric method, which coupled to its optical configuration, allows these low detection levels to be obtained reliably on a routine basis. The mercury vapour is transported into the detector through a chimney interface and is further sheathed in a stream of argon gas. This constrains the mercury in the argon stream and provides a rapid flush-out time between samples. The Merlin detector has effectively no cell, and the inherent problems associated with the traditional absorption cell devices, i.e. long flush-out times and non-linear responses, are thus overcome.

In co-operation with the University of Ghent, and by modification to its TouchStone software, P S Analytical has been able to produce a system for automated absolute calibration of mercury level. This is based on the work described by Dumarey *et al.* [3].

### *Injection of standard mercury vapour*

The argon gas inlet line has been modified to incorporate an Omnifit Septum introduction system Part No. 2505 PSA (Omnifit Ltd, 51 Norfolk Street, Cambridge CB1 2LE, UK). This allows a syringe to be inserted directly into the argon flow path to transport the mercury vapour directly over the gold-sand trapping unit. Prior to carrying out a calibration, the system has been optimized to provide the best peak shape and to minimize tailing. Checks have also been run to ensure that there is no remaining mercury on the trap once it has been revaporized.

The instrumentation should be assembled as shown in figure 3. The mercury vapour is injected into the Galahad, during the flushing stage of its operation, through the septum described above. The PSA

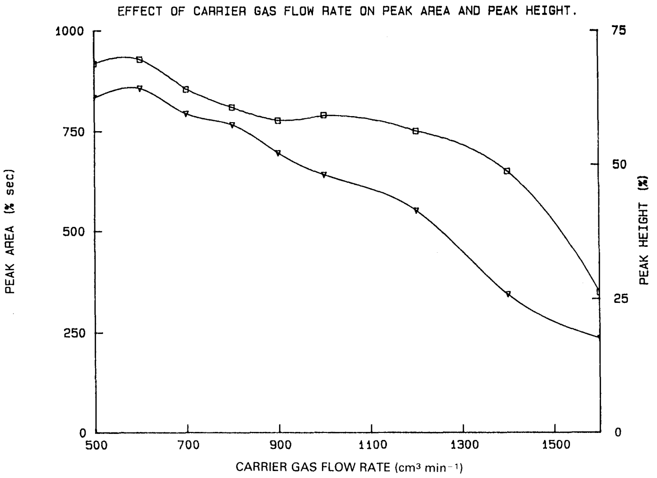

*Figure 4.*

TouchStone software guides the user through the various stages of the cycle.

The system is then optimized using a univariate approach.

*Effect of carrier gas flow rate on peak height/area/shape*

The instrument was set up with the following conditions: shield gas 400 ml/min, injection volume 30 µl, flush time 1 min, vaporize time 20 s and cooling time 1 min. Peak area/height measurements were then made with the argon gas flow ranging from 500–1600 ml/min. Figure 4 shows a plot of effect of flow rate on peak height and peak area. With increasing carrier gas flow rate, the peak area and heights both decrease. The optimum carrier gas flow rate was chosen to be 600 ml/min.

*Effect of shield gas flow rate on the peak height/area/shape*

With the same analytical conditions as above and the carrier gas set at 600 ml/min, the shield gas was varied over the range 0–600 ml/min. Figure 5 shows the plot of peak height and area against the flow rate. Clearly, a maximum level is obtained at 200 ml/min.

The above conditions were then subsequently chosen as the optimum operating conditions.

### Calibration of Galahad-Merlin combination

The calibration relies on the knowledge that, at a fixed temperature, the saturated vapour pressure of mercury is known and a fixed volume of vapour will contain a known quantity of mercury. This volume is absorbed onto the Galahad trap and then revaporized in to the Merlin Detector, where peak height and peak area are calculated. The PSA TouchStone Galahad version therefore has the necessary template to operate the Galahad, and also holds the calibration details. Once the temperature and volume are provided, the software computes the absolute level of mercury added to the trap.

To carry out these procedures reliably, close attention to detail is required; i.e. good housekeeping and a pure argon supply. An argon gas clean-up system is essential to ensure that any mercury in the argon is removed. A small vial and thermostatic bath are then also required to hold the mercury at a set temperature.

The calibration procedure is described here in a stepwise fashion, with specific references to the software interactions.

*Step 1:* Weigh 20–30 g of elemental mercury into a glass vial. Carefully transfer the closed vial to the thermostatic bath and monitor the temperature of the mercury vapour using a digital thermometer.

*Step 2:* Set up the equipment as explained in the installation procedure. Switch the instruments 'ON' and set the instrument parameters to the following conditions:

| | |
|---|---|
| *Carrier gas flow rate* | Argon 0·6 l $min^{-1}$ |
| *Shield gas flow rate* | Argon 0·2 l $min^{-1}$ |
| *Coolant gas* | Inert gas |

Note 1: Argon is recommended for the carrier and shield gas supply. Nitrogen and air reduce the sensitivity of the fluorescence detector by approximately eight and 30 times respectively. The coolant supply may be any inert gas but preferably argon which can then be operated from one argon supply.

*Galahad conditions*

*Flush time* = 30 s
*Vaporize time* = 20 s
*Coolant time* = 2 min

*Step 3:* Set up the computer and load the appropriate program.

Note 1: Any IBM compatible computer system can be used with the TouchStone software (P S

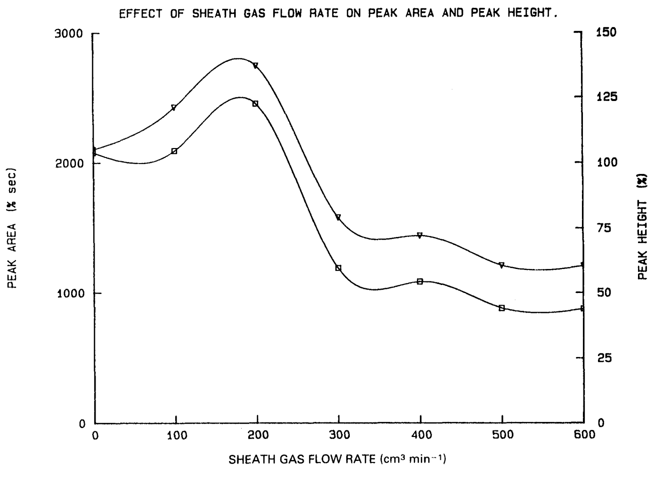

*Figure 5.*

```

Changed> TEST1.MTH - Hg-Gas Merlin+Galahad HG-G>Hg-Gas   Sample Tag   Ref Done

                                                          NO_TAG        0    1

 Library  Method  Calibrate  Options  Analyse  Results    5 Jul 89 11:23:09

Method: Hg-Gas Merlin+Galahad

Curve Fit by Equal Weight Slope Average

Measured by : Peak Ht

   FSA Merlin Calibration Setup:

RANGE 100      FINE max     ZERO Auto

Range & Running 1/4s set automatically

    Galahad Amalgam Trap Setup:
DELAY 1.0min  HEAT 10sec  COOL 1.0min

Gas Sampling Time 2  minutes

Gas Pump Flow Rate 0.5litres/min

Inert Gas:Argon 0.6litres/min

N.B.Master Method - Modify to Suit

        F1-Help  F2-Keep Displayed  F3-Print  F4-Edit  Esc-Menu

```

*Figure 6. Illustration of the TouchStone software methods page, providing the information required to operate the instrument combination to measure mercury in air.*

Analytical Ltd) to facilitate the calibration procedure.

Once the TouchStone program has been initialized, the menu page is displayed. To select an option use the cursor keys, or, alternatively, type the first letter of the option required and press 'RETURN'. The menu page can be readdressed using the 'ESCAPE' key.

*Step 4:* Follow the program instructions so that the appropriate program and method are displayed.

Note 1: The Galahad conditions are not computer controlled. The computer simply starts the Galahad cycle. However, if one wishes to alter the 'Gain' settings on the Merlin then this must be done by changing the conditions on the 'Methods' page, shown in figure 6.

*Step 5:* Return to the menu and then select the appropriate calibration requirements.

*Step 6:* Input the appropriate values (see figure 7 for a specific example).

Note 1: The saturation concentration of atomic mercury in air is calculated using the data of Weast [4] about the vapours pressures of mercury as a function of the temperature. The saturation

```

Galahad Trap Clean in Progress                           Sample Tag   Ref

                                                          NO_TAG       0    1

 Library  Method  Calibrate  Options  Analyse  Results    5 Jul 89 11:36:14

                                   New Calibration Curve

                              Galahad Absolute Calibration in ng/m3

                              2-Peak Ht 3-Pk Area? 2

                              No. of Standards (1-12) ? 5

                              No. of Runs per Std (1-12)? 5

                              Mercury Vapour Temperature? (C)20.0

                              Injection (μl):- Std1? 0.00

                              Injection (μl):- Std2? 10.00

                              Injection (μl):- Std3? 20.00

                              Injection (μl):- Std4? 30.00

                              Injection (μl):- Std5? 40.00

     F1-Help -Move Del-Delete BS)-Backdel ↵-Accept Esc-Menu

```

*Figure 7. Illustration of input required for a calibration curve, shown here are five injections with repeats. All are taken at 20°C. Before injecting standards the Galahad trap is automatically cleaned by heating.*

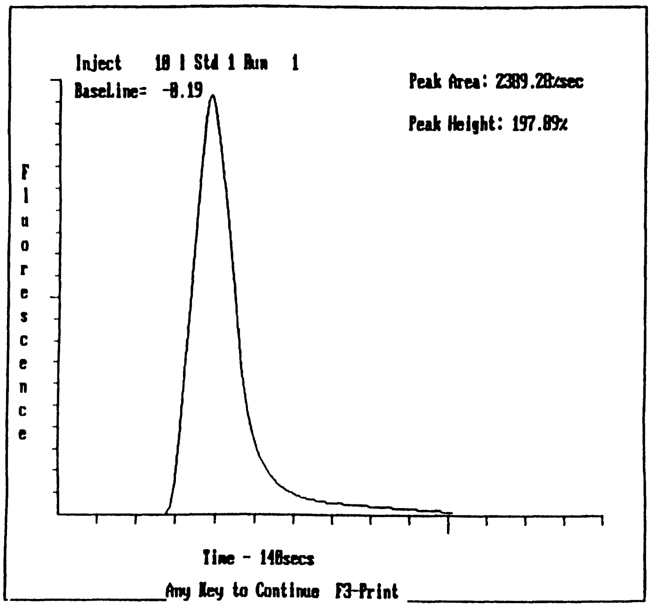

*Figure 8. Typical response from a volume of mercury adsorbed onto the gold-sand trap and revaporized into the Merlin detector.*

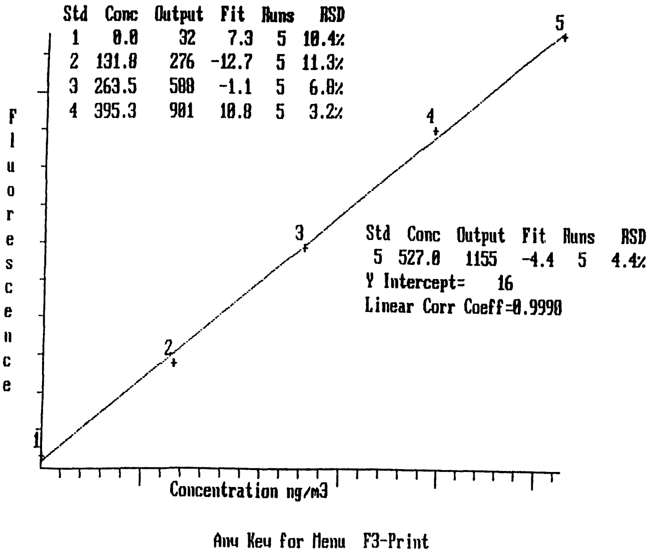

*Figure 9. Calibration graph obtained from injections etc. referred to in figure 7.*

concentration can be calculated using the formula:

$$C = \frac{3216522{\cdot}61}{T} \cdot 10^{-(A + B/T)}$$

Where: $C$ = Mercury concentration in air (ng/ml)
$A = -8{\cdot}134459741$
$B = 3240{\cdot}871534$
$T$ = Absolute temperature in Kelvin (= degrees Celsius + 273·16).

This calculation is performed automically by the software.

Note 2: The temperature can be varied in order to accommodate the use of various sizes of micro-syringe.

Note 3: When the last input has been entered, there will be an automatic 'Trap clean up'. When this is complete, the system is ready for the injection.

*Step 7:* Fill the syringe with the required volume of mercury vapour. Each gas micro-syringe will come with a set of instructions. Pressure locks on the micro-syringe ensure that no mercury vapour is lost before injection. Reproducibility of the signal may be improved by preconditioning the syringe. This is done by pulling in the maximum volume of saturated air, and leaving for 10 min, with the needle inside the flask.

*Step 8:* Activate the cycle using the computer and carefully inject the sample. When the run is complete, the computer will ask for the next standard to be injected.

*Step 9:* Repeat the procedure from Step 7 until you have obtained a calibration. A typical output is shown in figure 8 and a typical calibration graph obtained is shown in figure 9.

### Conclusions

The system described above provides a simple, reliable and effective means of calibrating a mercury absorption system in an absolute fashion. The fluorescence system is a very sensitive and accurate method of analysis. Samples

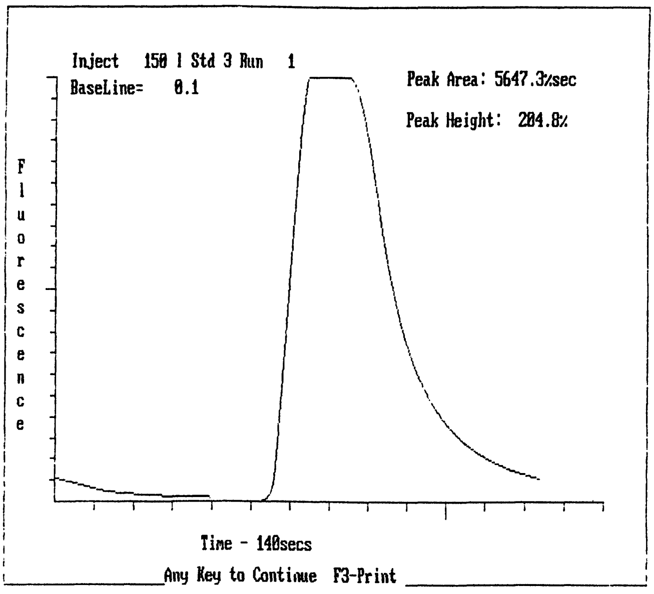

*Figure 10. Output signal obtained from Galahad/Merlin combination after collection on site for a 6 hour sampling period.*

of air-borne mercury can therefore be collected in a short time. Figure 10 shows an output of mercury from an absorption tube after collection at an urban site for several hours. This response is equivalent to more than 3000 ng of mercury. Clearly the system developed can provide good date after collection times of 10–30 min. Further work will be reported shortly describing the use of the system in the field.

### Acknowledgement

Original work on the use of the gold impregnated sand trapping system for mercury was carried out in the University of Gent, Proeftuinstraat 86, B-9000 Gent, Belgium. The cooperation of Professor C. Vandecasteele, Dr E. Temmerman and G. Vermeir is gratefully acknowledged. Warren T. Corns is supported by a SERC Studentship.

### References

1. Stockwell, P. B., Henson, A., Thompson, K. C., Temmerman, E. and Vandecasteele, C., *International Labmate.* **14**, II (1989), 45–47.

2. Temmerman, E. (in press).

3. Dumarey, R., Temmerman, E., Dams, R. and Hoste, J., *Analytica Chimica Acta*, **170** (1985), 337–340.

4. Weast, R. C. (ed.), *CRC Handbook of Chemistry and Physics*, 52nd edn (CRC Press, Cleveland, 1971).

